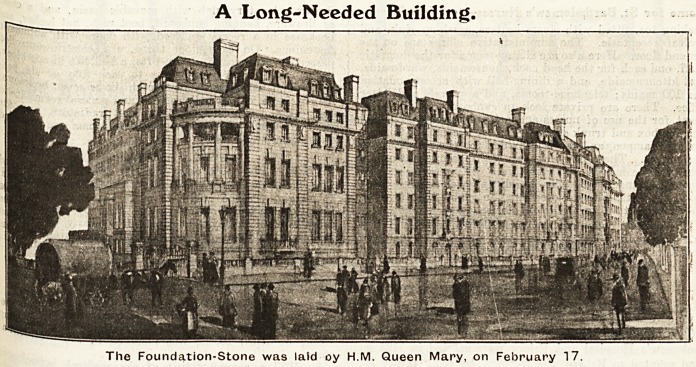# Queen Mary's Home for St. Bartholomew's Nurses

**Published:** 1921-02-19

**Authors:** 


### QUEEN MARY'S HOME FOR ST. BARTHOLOMEW'S NURSES

The nursing staff of St. Bartholomew's Hospital has waited long for adequate housing. The existing quarters comprise a number of old premises, formerly dwelling or business houses, which have from time to time been acquired by the hospital. They are scattered on various parts of the hospital site, are ancient, deficient in ordinary conveniences, costly as to service, difficult of supervision, and in every way totally unfitted for the purpose for which they are used. In the majority of cases two nurses have to share a room. In fact, there is no Nurses' Home at St. Bartholomew's, nor in the true sense of the word has the hospital at any time in its history had a Nurses' Home; for no part of the present quarters was built specially to provide accommodation for nurses. It is well known that for upwards of forty years the Governors have declared the provision of a Nurses' Home to be a first and most urgent need of the institution.

Now, at last, the first steps to give effect to this declaration have been completed. In 1919 the scheme for the provision of a new modern and adequate nurses' home was outlined and sanctioned, and the raising of the necessary funds began. Mr. McAdam Eccles, F.R.C.S., accepted the office of honorary secretary of the Nurses' Home Fund, and Mr. E. T. Mathews, O.B.E., F.R.I.B.A., architect of the hospital, was commissioned to prepare the plans for the building, Mr. E. T. Hall, F.R.I.B.A., acting as consulting architect. The first stage of their efforts was marked on the 17th inst.—after we go to press with this issue of The Hospital—when Her Majesty Queen Mary is to lay the foundation-stone of the first block of the new building, to be called Queen Mary's Home for St. Bartholomew's Nurses. The work on this block is to be proceeded with straight away, and it is anticipated that its erection will not occupy longer than eighteen months or two years. At the end of that period it is hoped that the funds for the second block (there are four blocks in all) may have been provided, so that the work may continue without interruption till the Home is complete.

### The Accommodation as Planned.

The building has been planned as four separate blocks, connected by enclosed bridges. Each block and each floor is self-contained, so that either can be isolated in case of necessity without interference with the remainder. Two of the blocks will be seven storeys, and the other two six storeys high. Our illustration gives an idea of the architectural features of the elevation; the external walls will be of Portland stone, with brick backing, with the floors, roofs, and supporting beams and piers in reinforced concrete. The buildings will occupy a triangular site at the south-east angle of the hospital, where most sunlight, air, and quiet can be obtained. The objects aimed at have been comfort, convenience, and economy in administration. The building is to be simple and practical, and extravagance will be avoided.

### All Single Bedrooms.

A feature of the Home is that there will be no double bedrooms. Each person—whether a member of the nursing or domestic staff—will have a separate bedroom. Five hundred and fifty-seven of these are provided, viz. for the matron, assistant matron, 48 sisters, 369 nurses, 5 hospital kitchen staff, 118 hospital and home maids, and 15 for the private nursing staff, the accommodation for whom is complete and entirely isolated in one block, with a separate entrance. There is a residential flat for the matron, a sitting-room for the assistant matron, one for the home superintendent, five sitting-rooms for sisters, who have also a common-room and a dining-hall; separate sitting-rooms, 16 feet high, for staff nurses and probationers, the former with a floor area of about 1,100 square feet and the latter about 600 square feet; a dining-hall with accommodation for 236 nurses; a recreation-room, 16 feet high, with a floor area of about 2,280 square feet, where a stage with retiring and dressing rooms is provided; a writing-room of about 600 square feet; a visitors' sitting-room; a lecture-room, examination-room, and library; and in the basement a small workroom for the use of nurses for dressmaking, and a laundry for their personal use which communicates with an ironing-room and drying-closet. On each floor of each of the three residential blocks are a room for shampooing, fitted with basins and electrical hair dryers, boot-rooms, cloak-rooms, and storage for boxes. Small rooms are provided for

**Home for St. Bartholomew's Nurses—(*continued*).**

making tea, fitted with steam heaters, wash-up sink, and tealeaf receptacle. The administrative offices are on the ground floor. Here also are sitting-rooms for the domestic staff, one each for the head cook, housemaids, wardmaids, and kitchenmaids, and a dining-hall with accommodation for 100 maids; telephone-rooms, and a parcels and letter office. There are private lock-up cupboards in the basement for the use of nurses and private nursing staff, and surplus box and trunk stores. Similar accommodation for boxes, shampoo, and tea is provided for the maids as for the nurses. There is a sick bay with accommodation for twenty-three nurses, a convalescents' sitting-room, and an open-air lounge. All the buildings are finished with asphalted flats, with enclosed sides to enable the nurses to spend some time in the open air.

The nurses' bedrooms are normally 12 feet by 8 feet in the clear, and it is intended that each shall have fixed furniture, consisting of dressing-table with glass and drawers, lavatory slab with movable basin, and a chest of drawers, hanging cupboard, boot rack, hat locker, and bookcase in a recess enclosed with doors with automatic fastenings. On each floor there are electric-controlled passenger lifts, coal and box lifts, a bed lift in one block, linen-rooms, water-closets—one to each five or six bedrooms, and bathrooms—one to each six or seven bedrooms. All corridors, dining-halls, offices, and general rooms are to be heated by hot-water pipes and radiators, and the sisters' sitting-rooms and bed-sitting rooms are provided with open fireplaces.

**What is now Wanted.**

The above is an outline of what the architect has planned and the Governors of St. Bartholomew's have sanctioned. It remains for the public to make an actuality of these intentions in the shortest possible time by providing the large sum of money that must of necessity be required for the building, equipment, and furnishing of this carefully-thought-out and admirable Nurses' Home.

## Figures and Tables

**Figure f1:**